# A Hybrid ViT-ECA-CF Framework with Adaptive Loss Reweighting for Imbalanced White Blood Cell Classification

**DOI:** 10.3390/bioengineering13101187

**Published:** 2026-10-12

**Authors:** Fouzia Jabeen, Jamal Hussain Shah, Maira Afzal, Muhammad Awais

**Affiliations:** 1 Department of Computer Science, COMSATS University Islamabad, Wah Campus, Wah Cantt 47040, Pakistan; 2 Department of Computer Science, College of Computer, Qassim University, Qassim 52571, Saudi Arabia

**Keywords:** vision transformer, deep learning, convolutional neural networks, WBC, efficient channel attention (ECA)

## Abstract

Background: White blood cells (WBCs), also known as leukocytes, play a central role in immune defense, and accurate WBC classification is essential for a hematological assessment and computer-assisted diagnosis. However, a severe class imbalance remains a major challenge in deep-learning-based WBC classification, particularly when minority classes contain substantially fewer training samples. Although data augmentation is commonly used to address this issue, synthetic transformations may fail to capture the complex morphological and appearance variations of WBCs and may introduce redundant patterns and additional computational costs. Methods: This study proposes a hybrid ViT-ECA-CF framework with adaptive loss reweighting (ALR) for minority-class WBC classification without data augmentation. The framework integrates efficient channel attention (ECA) to enhance discriminative feature representations and color-feature fusion (CF) to jointly capture morphological and staining-related color information from WBC components, including the nucleus, cytoplasm, and granules. An adaptive loss-reweighting strategy dynamically adjusts class contributions according to class distribution, prediction loss, and prediction confidence, thereby directing model optimization toward challenging minority WBC classes. Results: On the evaluated WBC classification dataset, the proposed framework achieved a 90.00% accuracy, 94.49% macro-F1, and 93.98% balanced accuracy, with an F1-score of 96.43% for minority classes. The framework substantially reduced the majority-to-minority performance gap, achieving a minority-to-majority F1 balance ratio of 1.053, indicating a comparable recognition performance across classes despite the severe imbalance. The proposed approach was also evaluated on independent datasets and compared with a CNN baseline in terms of training time. Conclusions: The findings provide an effective strategy by combining transformer-based feature representation, ECA, color-feature fusion, and adaptive loss reweighting to improve minority-class WBC recognition without relying on synthetic data augmentation. The proposed approach offers a potential strategy for handling a severe class imbalance. Further validation on larger and multicenter datasets is required to establish the robustness and clinical applicability of the proposed framework.

## 1. Introduction

White blood cell (WBC) classification is essential for the accurate and early diagnosis of immune and blood-related disorders. Despite significant improvements in deep learning models, the classification of white blood cells remains a challenging task [1]. The availability of balanced datasets for WBC classification is a major concern, as WBC datasets contain fewer images of basophils as compared to neutrophils. Class imbalance occurs when some classes contain substantially fewer samples than others. In the Raabin–WBC training set, basophils are the least represented class and are therefore treated as a minority class [2,3]. Similarly, lymphocytes and neutrophils contain more images as compared to eosinophils and monocytes [4,5]. This class imbalance can lead to lower classification performance with minority classes while achieving higher performance for majority classes, which can affect medical diagnosis. Missing minority-class cells in several conditions, such as chronic myeloid leukemia [6] and diseases related to eosinophils [7], can lead to delayed or incorrect diagnoses. Some traditional approaches such as data augmentation and resampling can address this class imbalance [8,9]. Data augmentation generates additional images by applying different transformation techniques to images, such as flipping, rotation, or adjusting the color. Recent studies have also shown that domain-specific augmentation can improve WBC classification performance. However, the effectiveness of these methods can vary depending on the characteristics of the WBC dataset and the applied transformations [10]. In WBC classification, conventional transformations such as scaling or rotating may not always provide substantial additional morphological information because these transformations generally preserve the underlying cellular morphology. Moreover, these methods increase training time and storage requirements, which often results in overfitting, specifically on noisy or small datasets. These limitations highlight the need for alternative methods that avoid data augmentation and provide solutions to improve model efficiency in recognizing the WBCs’ minority class. The multi-pipeline strategy allows the models to focus on the morphological features that are critical for the accurate classification of minority-class cells. The vision transformer with efficient channel attention and color-feature fusion model is proposed, and it achieves a 96.43% F1-score on minority classes without data augmentation, unlike traditional methods that apply robust augmentation [11] techniques on data. In cross-data validation, it also performed better compared with augmentation-based models, which exhibit a notable reduction in overall results. These findings indicate that the ViT-ECA-CF model with adaptive loss reweighting improves generalization across varying conditions of clinical images. The dataset used in this study includes five WBC classes with an imbalanced sample distribution, with basophils being the least represented class compared with neutrophils [5], which highlights the importance of class-balanced training. The proposed model captures fine-grained features such as granular texture, nuclear shape, and chromatin density factors, which are often ignored by standard augmentation methods. The integration of the attention mechanism with an adaptive loss reweighting strategy improves the model’s interpretability and robustness for white blood cell classification. Future work may include explainability modules to visualize attention maps and reinforcement gradients to strengthen clinicians’ confidence in decision-making. Overall, ViT-ECA-CF with adaptive loss reweighting provides a reliable, augmentation-free approach that improves the recognition of minority classes and enhances generalization in real-world hematology applications.

## 2. Literature Review

White blood cell (WBC) image classification is still hampered by the issue of class imbalance, which increases the risk of biased learning and decreases generalization. In medical image analysis, many datasets are imbalanced [12]. Some cell types are low-abundance classes, including basophils and eosinophils. This disparity results in a model that favors the majority classes, which increases the need for diagnostic tools. Existing deep network techniques and data augmentation techniques exhibit less effectiveness and fail to capture a specific morphology in medical datasets [13]. Recent research has shown a shift toward adjusting training weights to deal with class imbalance, rather than relying completely on data augmentation [14]. In recent studies, adaptive loss functions have been introduced [15], which help rebalance learning for minority classes and reduce the dominance of majority classes during model training [16].

Cost-sensitive learning is another method used for balancing [17]. It assigns a higher cost to misclassified samples of the minority class than to misclassifying samples from the majority class. This leads to more directed, focused learning for the minority class [18]. Transfer learning has also driven developments in imbalanced medical imaging [19], as models pre-trained on rich and diverse datasets can be fine-tuned on smaller, class imbalanced datasets while retaining essential visual features and adapting to domain specific requirements. Chen et al. [20] used transfer learning plus focal loss to increase the classification accuracy on minority classes without the addition of any data augmentation techniques. Models learn details using these strategies while addressing imbalance-training data. Although GAN-based augmentation can improve the representation of minority classes [12], precautionary measures are required when using synthetic data in sensitive medical applications. Recent studies have highlighted that synthetic data may introduce artifacts or affect the fidelity required for clinical decision-making and emphasize the importance of improving core model learning alongside the use of synthetic images [21]. In addition, attention guided convolutional networks [22] and reinforcement-aware learning strategies have been examined in the last couple of years to improve class detection. These models claim to learn to attend to the most informative regions in an image, while also adapting based on class-specific feedback as they are trained on the respective training dataset [23]. Their approach incorporated reinforcement-aware learning and encouraged the exploration of minority-class features, which was promising, especially when the visual patterns for the minority class were subtle in the dataset. This is especially relevant in WBC image classification, where there is little variability between classes. However, even slight variations can be clinically significant. Another promising development is the use of meta-learning strategies [24]. These strategies enable models to learn class-aware decision boundaries across various tasks. An author introduced a meta-learning approach that was able to quickly adapt to few shot scenarios and improve the accuracy of the minority class in highly imbalanced medical datasets [25]. These meta models reduce dependence on large, balanced datasets while optimizing learning based on the problem’s structure, even when limited labeled data is available, as often observed in the cases of clinical contexts. Cross-dataset validation has become a recent research focus to evaluate the generalization performance of imbalance-aware models.

Two models for WBC classification avoided augmentation and performed better on independent datasets, e.g., LISC, BCCD, and Raabin–WBC [26]. These models ensure a better accuracy with strong robustness and transferability, which are important requirements for a reliable diagnostic system. Furthermore, high accuracy alone is misleading if the classes are imbalanced, as it is often dominated by majority classes, making them less sensitive to minority classes. Researchers now use metrics such as the precision, recall, AUC, and F1-score to evaluate the performance more effectively on minority classes, rather than relying solely on accuracy [27]. A model that achieves a high accuracy may still fail to recognize rare disease conditions if minority classes are poorly represented. There has been a new emphasis on balancing the performance across all classes, not just the majority classes, especially in life-critical areas such as the detection of hematological diseases.

Kasprowicz and Silva [28] showed that vision transformer (ViT) models were compared with YOLOv11 variants for the classification of leukocyte classes using a private 14-class dataset, and the effect of HistAuGAN-based color augmentation was also evaluated. Similarly, Uddin et al. developed a lightweight model using Efficient Channel Attention (ECA) for five-class WBC classification, demonstrating that channel attention enhances the discriminatory power of features and the model was evaluated for robustness across different datasets with a lower computational cost [29]. Moreover, Ayesha and Khalidi proposed a self-attention-based Vision Transformer which was able to capture the long range dependencies in the blood cell images, producing a high classification accuracy [30]. Although these developments have taken place, current works are based on different strategies, including classical loss functions, fixed sampling approaches, data augmentation, attention mechanisms, and feature-based methods, to address class imbalance. However, the combined use of channel attention, handcrafted color features, and adaptive loss reweighting to tackle the class imbalance remains less explored. This constraint inspires the proposed ViT-ECA-CF with an Adaptive Loss Reweighting (ALR) framework to enhance the minority-class recognition ability while preserving a high overall classification accuracy. The literature shows that different strategies, including data augmentation, model benchmarking, and cross-dataset evaluation, have been explored for blood cell classification [31]. These studies do not establish a general superiority of augmentation-free methods over GAN- or SMOTE-based approaches. Rather, they indicate that different strategies may be suitable depending on the dataset and classification setting. Therefore, instead of assuming that augmentation is unnecessary, this study investigates an augmentation-free strategy that focuses on improving the model to learn distinguishing features from the original images. In summary, the literature reflects the use of both augmentation-based and augmentation-free approaches in handling a class imbalance in WBC image classification. This includes methods such as cost-sensitive loss, attention-guided learning, meta-learning, ensemble fusion, data augmentation, and cross-dataset validation. The overall results set the basis for reliable and clinically applicable models, motivating the current research to build an augmentation-free architecture that integrates channel attention, color feature fusion, and adaptive loss reweighting. The literature review is summarized in Table 1, which includes augmentation-based, transfer-learning, cost-sensitive, meta-learning, and attention-based methods.

This research addresses critical gaps in the existing research on performance transparency in imbalanced datasets. Previous studies have reported class-wise performance metrics, including F1 scores, for WBC classification. However, comparatively limited attention has been given to explicitly analyzing the balance of the F1 performance between majority and minority classes. This study is a clear example of displaying class-wise F1 score matrices, which provide detailed information about performance that may not be apparent from the overall accuracy. Rather than claiming that class-wise F1 reporting is absent from the literature, we introduce and report the F1 Balance Ratio to measure the evenness of the spread of performance across the majority and minority classes. One notable finding is that significantly improved performance was achieved by the proposed vision transformer variants. The F1 score obtained by these ViT variants for the minority class ranges from 56.52% to 96.43, showing that these variants can address a class imbalance more efficiently while improving the minority-class performance compared with existing CNN methods. Existing techniques may present a high overall accuracy without sufficiently emphasizing the balance of performance across individual classes. In contrast, this transformer architecture demonstrates the class-wise performance and balance between majority and minority classes. The majority–minority F1 balance ratio presents an additional quantitative measure of the balance between class-wise performance, providing complementary information to the class-wise F1 scores.

The proposed work addresses critical gaps in the existing methods. The literature shows the overall accuracy while the proposed work reports the class-level performance analysis. This study explicitly shows minority-class F1 scores and the balance ratio to analyze the performance equity across imbalanced classes. This study shows a substantial improvement in minority classes.

## 3. Research Gaps and Contributions

The main research gaps identified from the literature and the corresponding contributions of the proposed framework are summarized in Table 2. This comparison demonstrates how the proposed approach addresses the key limitations of existing methods.

The main contributions of this research are as follows:

- A novel strategy is proposed that replaces traditional augmentation to reduce feature redundancy and avoid training overload in the WBC dataset. The hybrid learning pipeline combines patch-level-feature fusion with adaptive loss reweighting to enhance model training efficiency, reduce redundancy, and provide focused learning from authentic image of white blood cells.
- An attention mechanism is used, which helps the network to focus on particular visual patterns of each class. A hybrid feature extraction method is used to extract the various morphological features according to the different types of WBCs, and optimization focuses on color features that are important for distinguishing overlapping or similar-looking cells.
- An Efficient Channel Attention (ECA) block is integrated to enhance feature robustness by emphasizing important channel-wise information. This reinforcement helps the model to learn discriminative patterns and increase its generalization capability and boost its accuracy, particularly for the complex and minority WBC classes.
- This paper proposed an Adaptive Confidence-Aware Loss Reweighting strategy, which dynamically adjusts class weights during training based on the class imbalance, difficulty of classification, and prediction confidence. The adaptive weights are integrated into the cross-entropy loss, enabling the model to emphasize underrepresented and difficult samples while preserving balanced learning across all classes.

## 4. Materials and Methods

**Figure 1:**
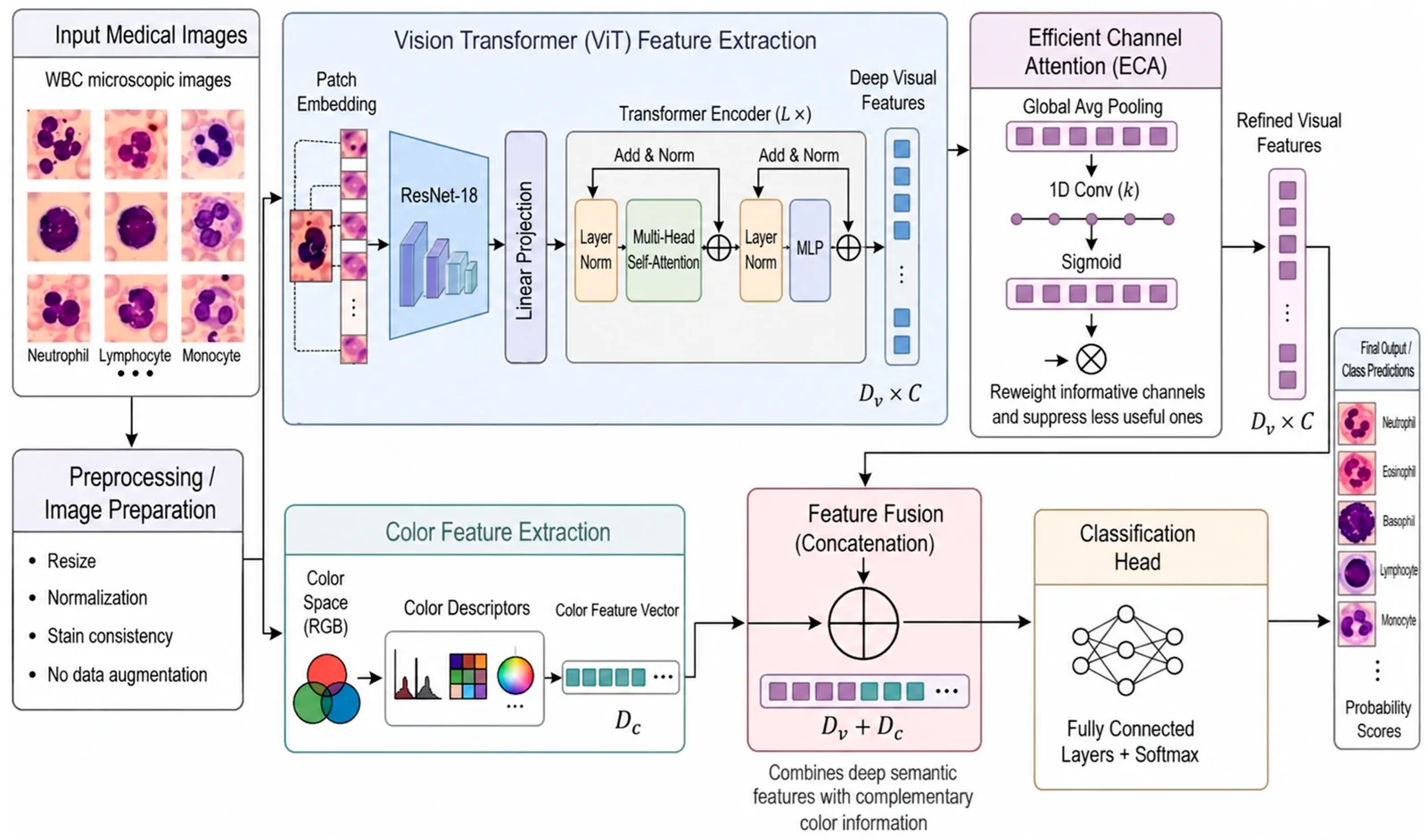
Proposed efficient channel attention and color feature fusion.

This research proposed a model to classify white blood cells while handling class imbalance by enhancing the model’s performance to accurately classify minority cell types without compromising its classification performance on majority cell types. First, each input image is processed and divided into appropriate patches that are then processed further by a dual-branch extractor. One branch extracts structural feature of the images using ResNet-18, which are further refined by Efficient Channel Attention (ECA) block to highlight important morphological patterns in the image. The second branch extracts color features from the image. The extracted features from both branches are combined to form a 515-dimensional token. This fusion ensures that both the low-level color variations and the high-level structural changes are properly utilized during training. The resulting fused patches are passed to a Vision Transformer (ViT) encoder, where a self-attention mechanism extracts further relationships between the patches. Furthermore, focal loss and weighted random sampling are used during training to handle the class imbalance problem, which prioritizes minority class samples without affecting the majority class. Overall, the step-by-step workflow is illustrated in Figure 1.

All input images are first resized to a uniform dimension of 224 by 224 pixels. This resolution maintains compatibility across models while also keeping the subcellular details intact. This standard sizing for images enables the model to train efficiently using batching and avoid scale-induced bias during patch tokenization or convolution. Various normalization techniques are applied to ensure consistency across the images. Moreover, each pixel $x_{i}$ in the image is rescaled to have a mean μ and standard deviation σ for the entire image as follows:

(1) $$x^{\prime }=\frac{x_{i}-\mu }{\sigma }$$

The changes ensure a consistent and similar distribution across the input channels (R, G, and B), improving the model convergence during training of the model. The resulting images are subsequently fed into the modified Vision Transformer [35] with feature extraction modules such as morphology, color, chromatics, and contextual reasoning, which are called Efficient Channel Attention and Color Fusion (ViT-ECA-CF). In the first step, model divides an input RGB image $I\;\epsilon \;R^{3\times H\times W}$ into non-overlapping patches of size p×p. Each image results in $N=\frac{H\cdot W}{p^{2}}$ patches. Each patch $P_{i}\in R^{3\times p\times p}$ retains local spatial and color information essential for WBC classification. Each patch is passed through a dual-branch feature extractor to extract features from five distinct classes of WBC. ResNet-18 is used due to its capacity to learn hierarchical spatial patterns in image data. Let the input image be represented by $X\in R^{H\times W\times C}$, where H, W, and C represent the height, width, and number of channels, respectively. The convolutional operation applied at layer l is mathematically defined as follows:

(2) $$F^{\left(l\right)}=\sigma \left(W^{\left(l\right)}*F^{\left(l-1\right)}+b^{\left(l\right)}\right)$$

where $F^{\left(l-1\right)}$ is the input feature map from the previous layer, $W^{\left(l\right)}$ represents the learnable convolutional kernel of size k×k, $b^{\left(l\right)}$ is the bias term, ∗ denotes the convolution operator, and σ is a non-linear activation function (typically ReLU). Each feature map captures spatially localized patterns that help distinguish between the morphological attributes of different WBC classes (e.g., nucleus shape, granularity, and texture). To reduce spatial dimensions and retain dominant features, a pooling operation—typically max pooling—is applied after selected convolutional layers, as shown below:

(3) $$P^{\left(l\right)}={MaxPool}_{s\times s}\left(F^{\left(l\right)}\right)$$

Here, s×s represents the window size.

The efficient channel attention (ECA) block is integrated into the proposed architecture without introducing more complexity in convolutional neural networks to enhance the capabilities of CNNs. Unlike existing methods that enhance attention using fully connected layers equipped with large number of parameters, the ECA block uses a local cross-channel interaction strategy to capture channel-wise dependencies. Let $X\;\epsilon \;R^{C\times H\times W}$ be the output feature map from a convolutional layer, where C is the number of channels, and H, W represent the spatial dimensions. The ECA block operates on global average pooling, Local Cross-Channel Interaction via 1D Convolution, and Channel-Wise Recalibration. Global average pooling is applied across the spatial dimensions to compress the feature map into a channel-wise descriptor, which can be represented as follows:

(4) $$z_{c}=\frac{1}{H\times W}\sum_{i=1}^{H}\sum_{j=1}^{W}X_{c}\left(i,j\right),\;\mathrm{for}\;c=\{1,2,\ldots ,C\}$$

This results in a vector $z\;\epsilon \;R^{C}$ representing the global statistics of each channel. Instead of using a fully connected layer, ECA introduces a 1D convolution with a kernel size k (an odd number, e.g., 3 or 5) to learn the attention weights across local channel neighborhoods, which is mathematical represented as follows:

(5) $$a=\sigma \left({\mathrm{Conv}1D}_{k}\left(z\right)\right)$$

where Conv1D_k_ is a 1D convolution of kernel size k, σ(·) denotes the sigmoid activation function, and a ∈ ℝ^C^ is the learned attention weight vector. The kernel size k is adaptively defined as follows:

(6) $$k=\psi \left(C\right)={\left|\log_{2}{\left(C\right)}^{\gamma }+b\right|}_{odd}$$

where γ and b are hyper-parameters (commonly γ = 2, b = 1), and the operation ${\left|\cdot \right|}_{odd}$ returns the nearest odd integer. The original feature map is reweighed by the attention vector through channel-wise multiplication: ${\hat{X}}_{c}=a_{c}\cdot X_{c},\;\mathrm{for}\;\mathrm{each}\;\mathrm{channel}\;c=1,2,\ldots ,C$, resulting in $\hat{X}\in R^{C\times H\times W}$, the refined feature map with enhanced informative channels and suppressed less-relevant ones. The final convolutional layer output $\hat{X}$ is flattened into a feature vector $z\in R^{512}$, where d denotes the total number of extracted features.

A dedicated branch for the color feature extraction process analyzes the input image in various color spaces to capture difference in the cytoplasm and nucleus of different types of white blood cells. This process helps to capture the morphological features of the image, which are difficult to capture using convolutional filters. Given an input RGB image $I\;\epsilon \;R^{H\times W\times 3}$, it is first converted into alternative color spaces such as HSV and Lab to extract perceptually relevant color components: $I^{HSV}=RGB2HSV\left(I\right),\;I^{Lab}=RGB2Lab\left(I\right).$ LAB is a perceptually uniform color space that separates luminance (L*) from color information (a*: green–red, b*: blue–yellow), mimicking human color perception. LAB gives Vision Transformers superior color discriminability and invariance to lighting variations by separating brightness from chrominance and using the attention mechanism to capture perceptually meaningful color differences rather than device-dependent RGB difference.

For each channel $I_{C}$, we compute first-order statistical descriptors to quantify the global color distribution: mean μ, standard deviation σ, skewness γ, and kurtosis $K_{c}$. Mean $\mu_{c}$ is defined as the mean intensity value for a specific color channel c (e.g., R, G, or B) in an image, which is calculated using as follows:

(7) $$\mu_{c}=\frac{1}{H\times W}\sum_{i=1}^{H}\sum_{j=1}^{W}I_{c}\left(i,j\right)$$

whereas standard deviation is computed by averaging all pixel values $Ic\;\left(i,j\right)$ across the spatial dimensions as shown below:

(8) $$\sigma_{c}=\sqrt{\frac{1}{H\times W}\sum_{i=1}^{H}\sum_{j=1}^{W}{\left(I_{c}\left(i,j\right)-\mu_{c}\right)}^{2}}$$

where H and W represent the image height and width for a given color channel c. Skewness $\gamma_{c}$ measures the spread or dispersion of pixel values $I_{c}\left(i,j\right)$ around the mean $\mu_{c}$ calculated across all spatial positions $\left(i,j\right)$ in an image of size H × W, defined as follows:

(9) $$\gamma_{c}=\frac{1}{H\times W}\sum_{i=1}^{H}\sum_{j=1}^{W}{\left(\frac{I_{c}\left(i,j\right)-\mu_{c}}{\sigma_{c}}\right)}^{3}$$

where skewness $\gamma_{c}$ represents the pixel intensity distribution for a color channel c, quantifying the asymmetry of the distribution. Kurtosis $K_{c}$ is calculated by averaging the fourth power of the normalized deviation of each pixel value $I_{c}\left(i,j\right)$ from the mean $\mu_{c}$, scaled by the standard deviation $\sigma_{c}$, over the entire image of size H × W as shown below:

(10) $$K_{c}=\frac{1}{H\times W}\sum_{i=1}^{H}\sum_{j=1}^{W}{\left(\frac{I_{c}\left(i,j\right)-\mu_{c}}{\sigma_{c}}\right)}^{4}$$

where $K_{c}$ defines the kurtosis of pixel intensities for a specific color channel c, which measures the tailedness or peakedness of the distribution; the histogram is computed by taking the average of the fourth power of the standardized pixel intensity deviations from the mean $\mu_{c}$ normalized by the standard deviation $\sigma_{c}$ across the entire image of size H × W. Histograms $H_{C}$ with fixed bin size b (e.g., b=16) are constructed for each color channel, defined as follows:

(11) $$H_{c}\left(k\right)=\frac{1}{H\times W}\sum_{i=1}^{H}\sum_{j=1}^{W}\delta_{k}\left(I_{c}\left(i,j\right)\right),\ldots with\;k\;\in \left\{1,2,\ldots ,\;K\right\}$$

where δ_k_ (·) is an indicator function that returns 1 if the pixel intensity falls within the kth bin range, and 0 otherwise. The extracted features from all color channels are concatenated to form a comprehensive high-dimensional color feature vector $F_{raw}=\left[\mu_{R},\;\sigma_{R},\;\gamma_{R},\;K_{R},\ldots \ldots \ldots ,\;\mu_{b},\;\sigma_{b},\;\gamma_{b},\;K_{b}\right]$. Given nine channels across RGB, HSV, and Lab, with four statistical descriptors and b histogram bins per channel, the resulting feature dimension is $D_{raw}=9(4+b)$. This high-dimensional color feature vector is passed through a fully connected layer to obtain a compact 3-dimensional color representation F_color_ ∈ $R^{3}$. The resulting 3-dimensional color representation is then concatenated in parallel with the 512-dimensional CNN feature representation to produce a 515-dimensional patch token, $F_{patch}=\left[F_{ResNet};\;F_{color}\right]\in R^{515},$ which represents both structural and color features.

A learnable [CLS] token $e_{cls}\in R^{d}$ is inserted at the beginning of this resulting sequence of patch tokens, where $d=d_{r}+3.$ This token serves as the global representation for classification. Additionally, a learnable positional encoding $PE\;\epsilon \;R^{\left(N+1\right)\times 515}$ is added to the token sequence to preserve spatial relationship as follows:

(12) $$Z=\left[e_{cls};\;e_{1};\;e_{2};\ldots ;e_{N}\right]+PE$$

This enriched sequence is passed into the Vision Transformer (ViT) Encoder, which contains a multi-head self-attention layer and a feedforward layer to capture long-range dependencies across patches and global information in [CLS] tokens. Each patch token has a dimension of 515, obtained by concatenating the 512-dimensional CNN feature vector with the 3-dimensional color feature vector. The final [CLS] token output (a vector of dimension d = 515) is passed into a fully connected (linear) layer to produce predicted logits for each class, $z=W_{cls}\;h_{CLS}+b_{cls},$ where $h_{CLS}\in R^{512}$ is the final [CLS] representation from the encoder, $W\in R^{C\times d}$ is the weight matrix of the classifier, $b_{cls}\in R^{C}$ is the classifier bias, and $z\in R^{C}$ are the raw logits for $C_{WBC}$ classes.

A confidence-aware adaptive loss reweighting strategy is introduced to dynamically adjust the contribution of each class during model optimization. Unlike conventional weighted Cross-Entropy loss, where class weights are computed once based on the training distribution and remain fixed throughout the learning process, the proposed approach continuously updates class weights according to the learning behavior of the network. The adaptive mechanism considers three factors: (1) class distribution, (2) class-wise learning difficulty, and (3) prediction confidence. This enables the model to automatically allocate greater importance to underrepresented and difficult-to-classify WBC categories.

Initially, class weights are calculated based on the number of samples available in each class. For a dataset containing C classes, the initial weight for class c is defined as follows:

(13) $$w_{c}^{0}={\left(\frac{N_{max}}{N_{c}}\right)}^{\alpha }$$

where $N_{c}$ represents the number of training samples in class c, $N_{max}$ denotes the sample count of the majority class, and α is a scaling parameter controlling the impact of class imbalance.

During training, the ViT-ECA model generates predictions for each input image, and class-wise loss and prediction confidence are recorded. The average loss of each class represents the learning difficulty, while the average confidence indicates how reliably the model recognizes that class. Based on these parameters, the class weights are dynamically updated after each epoch as follows:

(14) $$w_{c}^{\left(t+1\right)}=w_{c}^{t}\left(1+\beta {\bar{L_{c}}}^{\left(t\right)}\right)\left(1+\gamma \left(1-{\bar{P_{c}}}^{\left(t\right)}\right)\right)$$

where $w_{c}^{\left(t+1\right)}$ is the updated weight for class c at epoch t+1, ${\bar{L_{c}}}^{\left(t\right)}$ represents the average classification loss of class c during epoch t, and ${\bar{P_{c}}}^{\left(t\right)}$ denotes the average prediction confidence for correctly classified samples of class c. The parameters β and γ control the contribution of classification difficulty and prediction uncertainty, respectively. The values of β and γ are set to 0.5, weight minimum is 0.2, and weight maximum is 5.

The dynamically updated weights are incorporated into the Cross-Entropy objective function:

(15) $$L_{adaptive}=-\sum_{c=1}^{C}w_{c}^{\left(t\right)}y_{c}log\left(p_{c}\right)$$

where $y_{c}$ represents the ground-truth label and $p_{c}$ denotes the predicted probability for class c.

During each training epoch, the model first learns discriminative features through the ViT-ECA architecture. The obtained predictions are then used to compute class-wise loss and confidence statistics. At the end of every epoch, the proposed adaptive module recalibrates the class weights, which are subsequently used in the next optimization cycle. Therefore, the proposed strategy allows the model to progressively focus on challenging minority classes while reducing unnecessary emphasis on already well-learned majority classes. The complete optimization procedure is summarized in Algorithm 1.
**Algorithm 1:** Adaptive Loss Reweighting for ViT-ECA Optimization**Input:** Training dataset $D={\left\{\left(x_{i},y_{i}\right)\right\}}_{i=1}^{N}$, Number of classes C, ViT-ECA model parameters θ, Number of training epochs T, Hyperparameters α,β,γ **Output:** Optimized ViT-ECA model parameters $\theta^{*}$
**Step 1: Initial Class Weight Computation**
       1.Calculate the number of samples $N_{c}$ for each class c, where c∈{1,2,…,C}.       2.Compute initial class weights using class distribution: $w_{c}^{0}={\left(\frac{N_{max}}{N_{c}}\right)}^{\alpha }$where $N_{max}$ represents the number of samples in the majority class.**Step 2: Model Training**       3.Initialize ViT-ECA network parameters θ.       4.For each epoch t=1 to T: 
     **a. Mini-batch Processing**       5.For each mini batch B from training dataset:             i. Input images $x_{i}$ into the ViT-ECA network. ${\hat{y}}_{i}=f\left(x_{i};\theta \right)$             ii. Obtain predicted probabilities $p_{i}$ for each class.             iii. Compute weighted classification loss: $L_{ALR}=-\sum_{c=1}^{C}w_{c}^{t}y_{c}log\left(p_{c}\right)$             iv. Perform backpropagation and update model parameters: $\theta =\theta -\eta \nabla_{\theta }L_{ALR}$where η represents the learning rate.**Step 3: Adaptive Class Weight Updating**       6.After completing each epoch, calculate classwise training statistics:           i. Average classification loss: ${\bar{L_{c}}}^{t}$           ii. Average prediction confidence: ${\bar{P_{c}}}^{t}$ for each class c.       7.Update class weights dynamically: $w_{c}^{t+1}=w_{c}^{t}\left(1+\beta {\bar{L_{c}}}^{t}\right)\left(1+\gamma \left(1-{\bar{P_{c}}}^{t}\right)\right)$where:         •β controls the effect of class difficulty,          •γ controls the effect of prediction uncertainty.
**Step 4: Repeat Optimization**       8.Use the updated class weights $w_{c}^{t+1}$ for the next epoch.       9.Repeat Steps 4–8 until the maximum number of epochs T is reached.       10.Return the optimized ViT-ECA model $\theta^{*}$.


## 5. Results and Discussion

A comparative analysis of the proposed ViT-ECA-CF model and seven existing CNN models using imbalanced dataset is presented in this section. The results are obtained in terms of Precision, Recall, Accuracy, and F1 score. Additionally, the performance of each class separately, ablation studies, and Grad Cam visualizations are also presented to show the model’s effectiveness and interpretability. The experimental setup used in this research includes the workstation having an NVIDIA GeForce RTX 3060 GPU, 12 GB VRAM, 32 GB DDR4 RAM, and Intel Core i5-12400F processor, transformer architectures, scikit-learn used for performance evaluation, and PIL for image handling. In addition, a modified Vision Transformer was employed that used ResNet-18 as the patch feature extractor, followed by ECA and Transformer-based feature processing. Input WBC images were resized to 224 × 224 pixels and partitioned into 16 × 16 non-overlapping patches, resulting in 196 patch tokens. The learnable class (CLS) token along with the learnable positional embeddings was appended before Transformer encoding. The Transformer encoder consisted of 12 encoder layers, each comprising 12 multi-head self-attention heads with a head dimension of 64, followed by a feed-forward network with a hidden dimension of 3072 and GELU activation. Pre-Layer Normalization and residual skip connections were employed throughout the encoder blocks. To enhance discriminative channel-wise feature representation, an Efficient Channel Attention (ECA) module was incorporated to enhance the discriminative channel-wise feature representation with its adaptive kernel selection parameters γ = 2 and b = 1, resulting in a 1D convolution kernel size of 5, followed by sigmoid activation to generate channel attention weights.

The model was initialized with ImageNet pretrained weights and optimized using the Adam optimizer with a learning rate of 1 × 10^−4^. Training was performed for 20 epochs using a batch size of 32. To compare the impact of different loss functions on class imbalance learning, the proposed architecture was trained using cross-entropy, weighted cross-entropy, and focal loss (γ = 2.0). The proposed adaptive loss reweighting function initializes class weights based on the inverse class frequency computed from the training set. These weights are subsequently updated after each training epoch using class-wise learning difficulty and prediction confidence, allowing the weighted Cross-Entropy loss to dynamically adjust the contribution of each class during optimization. The proposed ViT-ECA framework was primarily trained and evaluated using the Raabin-WBC dataset [5] which has high-resolution microscopic images. The peripheral blood smears shown in the dataset are classified into five main categories of leukocytes presented in Table 3.

As the Raabin–WBC dataset provides predefined training and testing partitions, *k*-fold cross-validation was performed on the predefined training set. The model was trained using the predefined training set within the cross-validation procedure, while Test-A and Test-B were kept separate and used only for evaluation, ensuring consistency with the original dataset protocol. To assess the generalization capability of the proposed framework, an independent evaluation was conducted using the predefined Test-B subset of the Raabin–WBC dataset. Test-B contains double-labeled neutrophil and lymphocyte images acquired under different imaging conditions from the training data, making it suitable for assessing robustness under an unseen acquisition setting. The Test-B images were not used during cross-validation, model training, or model selection, and served solely as an independent evaluation set. To further validate the proposed framework, experiments were also performed on the LISC dataset. Since LISC is primarily designed for leukocyte segmentation and classification, the white blood cells were first cropped from the original images using the available annotations before being provided as input to the trained ViT-ECA model. The LISC dataset was used exclusively for external validation, and no images from this dataset were included during cross-validation, training, or model selection.

**Figure 2:**
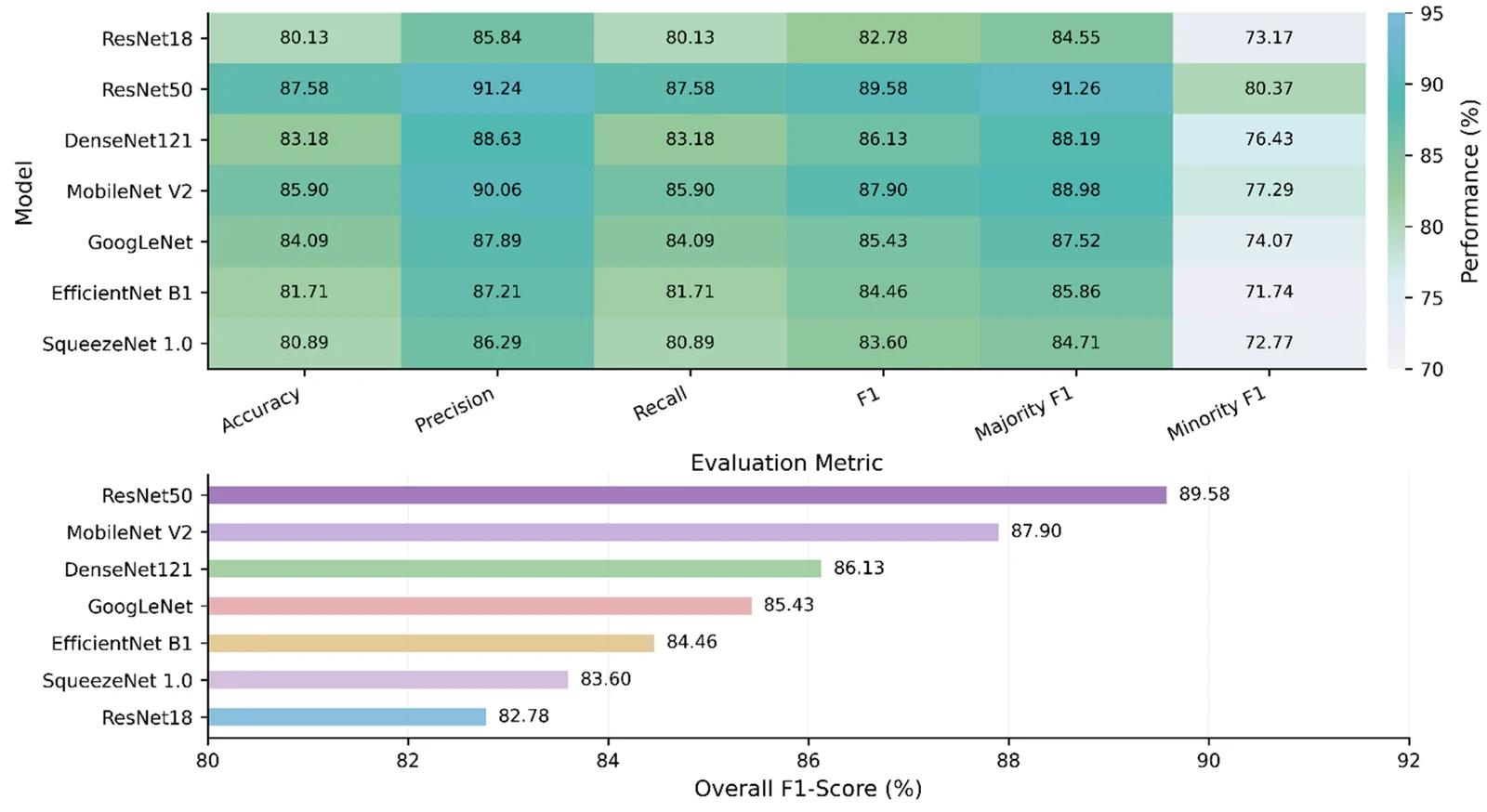
Comprehensive performance comparison: annotated metric heatmap and F1-score ranking.

To assess the robustness and reproducibility of the proposed framework, the comparative experiments between the strongest baseline (ResNet50) and the proposed ViT-ECA-CF model were repeated five times using different random initialization seeds while maintaining identical data splits and hyperparameter settings. The results are reported as mean ± standard deviation (SD). Statistical significance was evaluated using the Wilcoxon signed-rank test, with *p* < 0.05 considered statistically significant. Table 4 provides a summary of the class-wise performance of the tested baseline CNN architectures on the imbalanced WBC dataset, without any data augmentation, offering a summarized comparison of overall performance, F1-scores, and the majority–minority class behavior. Furthermore, this table provides a quantitative standardization of the trends in comparison above, offering insight into the performance differences among architectures and cell types under the same training conditions. Additionally, Figure 2 shows the comprehensive model performance comparison using metric heatmaps and F1-score rankings.

Table 5 presents the results of the ablation study on the proposed ViT-ECA-CF model, which isolates the impact of each architectural and training mechanism on performance by class and overall. The table serves as a quantitative reference in a structured format, outlining successive design decisions regarding the model’s behavior under the same experimental conditions.

**Figure 3:**
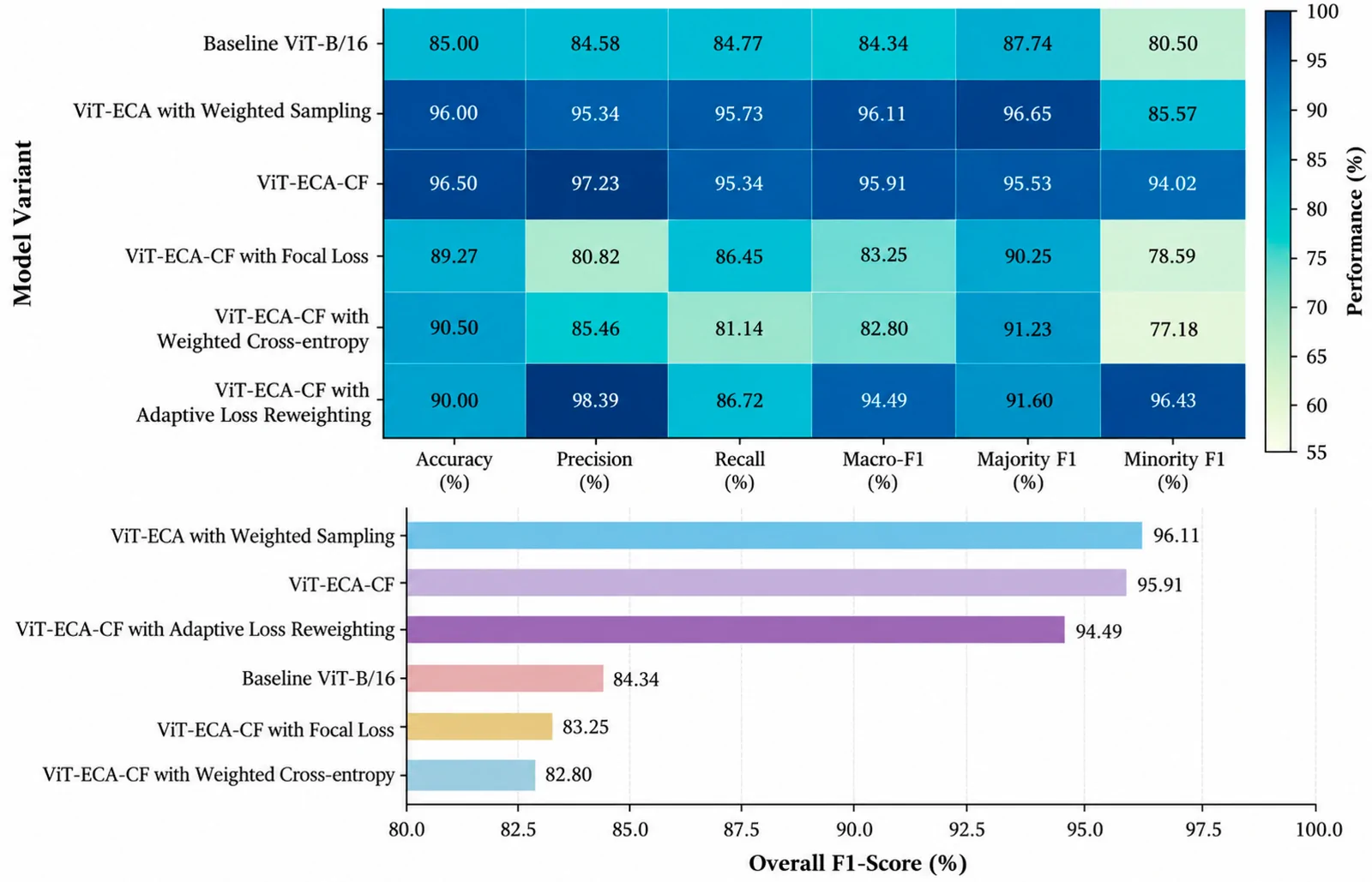
Overall performance comparison of the evaluated ViT model variants.

In addition, Figure 3 shows the proposed variant demonstrates a balanced overall classification performance.

Table 6 and Table 7 provide a consolidated comparison between CNN baselines and proposed ViT-based architectures. Among CNN models, ResNet50 achieves the highest performance with 87.58% accuracy and 89.58% F1-score but maintains a 10.89% majority-minority gap. The proposed ViT-ECA-CF achieves the highest accuracy (96.50%) with a minimal gap of 1.51%.

The gap for ViT-ECA-CF with adaptive loss reweighting is inverted to −4.83%, with an F1-score of 96.43% for minority classes, which is the best among all considered models. The results show that architectural innovation (ECA and CF) is an effective solution for the class imbalance problem in WBC classification. Incorporating ECA maintains competitive accuracy while providing a better handling of class attention, although minority classes remain slightly weaker. Further, adding color features significantly improves basophil recognition and enhances class-specific representation, even though the overall accuracy decreases. The use of focal loss further balances class weights, resulting in a better recall for minority classes and fair performance across classes. The complete ViT-ECA-CF model not only provides stability for majority classes but also enhances the recognition performance of minority classes and obtains a well-balanced trade-off. Finally, the use of weighted sampling and Adaptive Loss Reweighting gives a significantly large improvement in the F1 score of the minority class, along with a high performance for the majority class, thus contributing to strong generalization and proving the effectiveness of the proposed approach for solving the class imbalance. Table 8 presents a comprehensive performance comparison of existing CNN architectures and the proposed ViT-based variants, evaluated across accuracy, precision, recall, and F1-score metrics, with a specific emphasis on the performance disparities between majority and minority classes. The proposed ViT-based framework substantially enhances minority-class detection on severely imbalanced datasets. By incorporating ECA attention, color features, focal loss, weighted sampling, and adaptive loss, model variants achieve significant improvements across all metrics. Compared to the best-performing CNN baseline (ResNet50, 80.37% minority F1-score), ViT-ECA-CF with weighted sampling and Adaptive Loss Reweighting achieves a 96.43% minority F1-score, representing a 16.06 absolute improvement. Concurrently, the framework achieves an overall F1-score of 94.49%, an accuracy of 90.00%, a precision of 98.39%, and a recall of 86.72%, demonstrating a well-balanced classification performance. These results confirm that the proposed approach effectively addresses extreme class imbalance while maintaining strong performance across both minority and majority classes. The F1 balance ratio in Table 8 is calculated using the formula given in Equation (16).

(16) $$F1\;Balance\;Ratio=\frac{Minority\;Class\;F1}{Majority\;Class\;F1}$$

To further investigate the feature learning capability of the proposed framework, Table 9 compares the Grad-CAM activation maps of ResNet-50 and the proposed ViT-ECA model.

**Figure 4:**
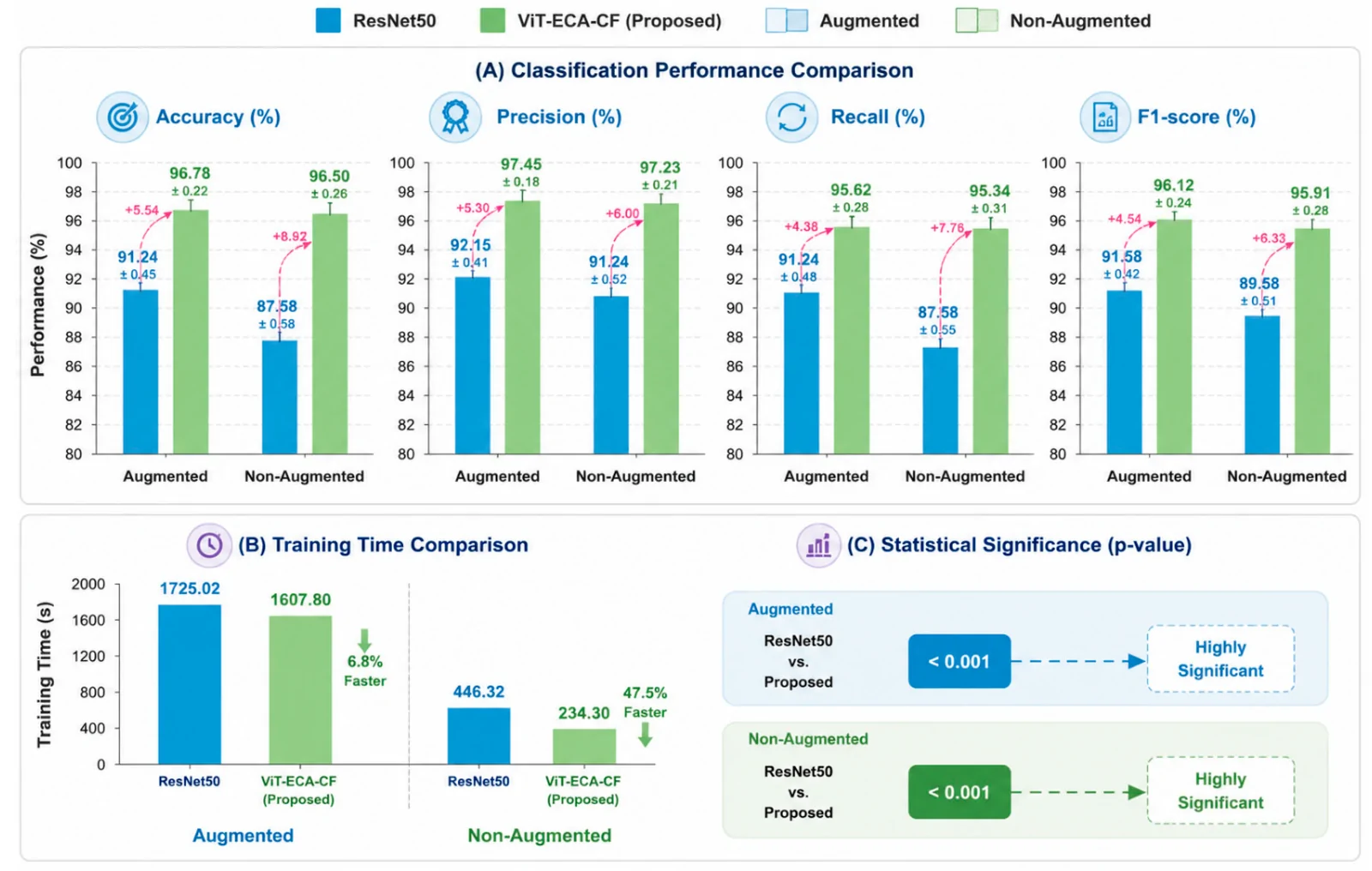
Comparative evaluation model under augmented and non-augmented dataset settings.

Table 10 provides a comparative analysis of the proposed model with the best existing model, using both augmented (random rotation, vertical/horizontal flip, random cropping and resizing, contrast adjustment, and color jitter) and non-augmented data. The proposed model achieves a very high precision, recall, and F1-scores, and its classification performance is strong and reliable. The results demonstrate that the proposed method improves predictive performance while reducing computational cost, especially in the non-augmented setting. In addition, the proposed ViT-ECA-CF model consistently outperformed the strongest CNN baseline (ResNet50) under both augmented and non-augmented settings. The low standard deviations across fifteen independent runs indicate stable and reproducible performance, while the Wilcoxon signed-rank test confirmed that the observed improvements were statistically significant (*p* < 0.05). Figure 4 shows the graphical representation of the augmented and non-augmented dataset settings.

Table 11 shows the proposed model performance on LISC dataset. The reduction in performance on LISC is primarily attributable to a cross-dataset distribution shift, including differences in imaging conditions, staining characteristics, and cellular morphology. Despite these variations, the model maintained relatively strong recall and Macro-F1, indicating that the learned discriminative representations retain a substantial generalization capability on an independent dataset.

The proposed framework is compared with recent state-of-the-art methods in terms of computational complexity and robustness in Table 12. With 85.8 M parameters and 33.6 G FLOPs, the proposed model gives competitive computational efficiency among the compared approaches and achieves the lowest inference time (8.8 ms), making it suitable for real-time clinical applications. Moreover, the proposed framework demonstrates good generalization ability when tested on the Raabin–WBC, Test-B, and LISC datasets, demonstrating its robustness in various imaging conditions.

## 6. Conclusions and Future Directions

A Vision-Transformer-based Efficient Channel Attention with Adaptive Loss Reweighting framework is proposed in this research for the classification of minority white blood cell classes under severe class imbalance. The proposed technique enhances minority class learning by combining the global representation capability of ViT with Efficient Channel Attention and adaptive loss reweighting, thereby preserving the original data distribution while avoiding data augmentation. Experimental results show that the proposed technique achieved a 96.43% F1 score of the minority class, significantly improved minority-class recognition, reduced the performance gap between majority and minority classes, and showed an improved minority-class performance compared with the selected CNN baselines. A cross-dataset evaluation further demonstrated the robustness and generalization capability of the proposed framework across different imaging conditions. Additionally, the evaluation on Test-B and LISC provided additional evidence of the model performance under different imaging conditions. Although these results are promising, prospective clinical validation on diverse patient populations is still required before routine real-world clinical deployment. Grad-CAM visualizations provided qualitative insight into the regions contributing to the model predictions. Despite the promising performance of the proposed framework, several limitations remain. The model may experience reduced performance on low-quality images, staining variations, imaging artifacts, overlapping cells, or rare white blood cell morphologies that are underrepresented in the training data. In addition, visually similar leukocyte subtypes with subtle morphological differences may still be challenging to distinguish, potentially leading to misclassification. Further validation on larger multicenter clinical datasets, including prospective evaluation and patient-level analysis, is required before assessing clinical applicability. Future work will investigate more advanced adaptive loss optimization and hybrid data-balancing strategies to further enhance the complementary strengths of adaptive loss reweighting and sampling under extreme class imbalance. The framework will also be extended to classify additional white blood cell subtypes using larger multicenter datasets. Furthermore, lightweight transformer architectures, multimodal clinical information, enhanced explainability techniques, and prospective multicenter clinical validation will be explored to improve the real-world reliability and clinical applicability of the proposed framework.

## Figures and Tables

**Table 1:** Comparative summary of the literature review.

Ref.	Method/Approach	Main Contribution	Advantages	Limitations	Gap Addressed by Proposed Work
[14]	Unified loss function	Unified loss for handling class imbalance	Improves minority learning without synthetic data	Designed for segmentation rather than classification	Motivates adaptive loss reweighting for WBC classification
[15]	Adaptive voxel-weighted loss	Adaptive loss for prostate lesion detection	Better minority-class recognition	Limited to PET/CT imaging	Demonstrates the benefit of adaptive loss for medical imaging
[17]	Cost-sensitive DNN	Cost-sensitive learning for brain tumor classification	Reduces minority misclassification	Fixed cost weights; MRI-specific	Supports adaptive rather than static loss weighting
[32]	Deep transfer learning	WBC classification without augmentation	Avoid synthetic image generation	Limited feature discrimination for minority classes	Supports augmentation-free learning strategy
[21]	Synthetic data review	Review of GAN-based medical image augmentation	Increases data diversity	May introduce unrealistic clinical features	Justifies avoiding synthetic augmentation
[22]	Attention-guided YOLOX	Attention-based blood cell detection	Improves localization and feature extraction	Detection only; computationally expensive	Motivates lightweight attention mechanism
[33]	TXL-PBC dataset	Public WBC dataset with cross-dataset evaluation	Demonstrates model generalization	Limited benchmarking across diverse datasets	Supports cross-dataset validation
[27]	CNN benchmark study	Performance comparison of multiple CNNs	Provides strong baseline comparison	No architectural innovation	Justifies baseline selection and evaluation metrics
[28]	Vision Transformer (ViT)	ViT for leukocyte classification	Better global feature representation	Relies on color augmentation	Supports ViT backbone without augmentation
[29]	Efficient Channel Attention (ECA)	Lightweight channel attention	Improves feature representation with low computational cost	Does not explicitly address class imbalance	Motivates ECA module in proposed framework
[34]	Vision Transformer	Self-attention for blood cell classification	Captures long-range dependencies	Computationally intensive; imbalance remains	Supports transformer-based feature extraction
[31]	DAFFNet	Dual-attention feature fusion	Improves fine-grained WBC recognition	No adaptive imbalance handling	Motivates combining attention with adaptive loss

**Table 2:** Summary of research gaps addressed and proposed contributions.

Research Gap	Proposed Contribution
Minority-class performance not reported	Explicit minority-class evaluation
Lack of balanced-performance metrics	Minority–majority F1 balance analysis
Poor recognition of rare classes	Hybrid ViT-ECA with adaptive loss reweighting
Dependence on augmentation	No augmentation required
Limited external validation	Cross-dataset validation

**Table 3:** Data description and description of minority and majority classes.

Images	Class	Number of Images	Percentage Out of Total	Types
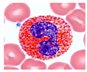	Eosinophil	322	7.42%	Minority
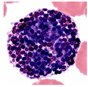	Basophil	89	2.05%	Minority
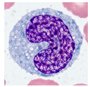	Monocyte	234	5.39%	Minority
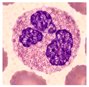	Neutrophil	2660	61.31%	Majority
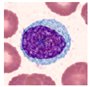	Lymphocyte	1034	23.83%	Majority

**Table 4:** Class-wise performance comparison of baseline CNN architectures on the imbalanced WBC dataset without data augmentation.

Model	WBC Class-Wise Comparison				Overall Performance
	Class	Precision (%)	Recall (%)	F1-Score (%)	
ResNet18	Neutrophil	88.00	83.00	85.40	Accuracy (80.13%) Precision (85.84%) Recall (80.13%) F1 (82.78%) Majority F1 (84.55%) Minority F1 (73.17%) **Analysis:** Improved baseline but still weaker on minority cells.
	Lymphocyte	86.00	79.00	82.40	
	Eosinophil	78.00	70.00	73.70	
	Basophil	74.00	68.00	70.90	
	Monocyte	75.00	72.00	73.30	
ResNet50	Neutrophil	94.00	90.00	92.00	Accuracy (87.58%) Precision (91.24%) Recall (87.58%) F1 (89.58%) Majority F1 (91.26%) Minority F1 (80.37%) **Analysis:** Performs best overall among CNNs; minority gap reduced.
	Lymphocyte	92.00	87.00	89.40	
	Eosinophil	84.00	78.00	80.90	
	Basophil	80.00	75.00	77.40	
	Monocyte	83.00	79.00	80.90	
DenseNet121	Neutrophil	91.00	87.00	88.90	Accuracy (83.18%) Precision (88.63%) Recall (83.18%) F1 (86.13%) Majority F1 (88.19%) Minority F1 (76.43%) **Analysis:** Balanced, slightly conservative predictions; stable minority results.
	Lymphocyte	89.00	84.00	86.40	
	Eosinophil	80.00	75.00	77.40	
	Basophil	74.00	70.00	71.90	
	Monocyte	78.00	74.00	75.90	
MobileNet V2	Neutrophil	92.00	87.00	89.40	Accuracy (85.90%) Precision (90.06%) Recall (85.90%) F1 (87.90%) Majority F1 (88.98%) Minority F1 (77.29%) **Analysis:** Lightweight with near-ResNet50 accuracy; efficient and consistent.
	Lymphocyte	90.00	86.00	87.90	
	Eosinophil	81.00	75.00	77.90	
	Basophil	77.00	71.00	73.90	
	Monocyte	79.00	75.00	77.00	
GoogLeNet	Neutrophil	91.00	86.00	88.40	Accuracy (84.09%) Precision (87.89%) Recall (84.09%) F1 (85.43%) Majority F1 (87.52%) Minority F1 (74.07%) **Analysis:** Performs reliably with slightly lower recall; good efficiency.
	Lymphocyte	88.00	83.00	85.40	
	Eosinophil	78.00	72.00	74.90	
	Basophil	72.00	68.00	69.90	
	Monocyte	76.00	72.00	73.90	
EfficientNet B1	Neutrophil	89.00	84.00	86.40	Accuracy (81.71%) Precision (87.21%) Recall (81.71%) F1 (84.46%) Majority F1 (85.86%) Minority F1 (71.74%) **Analysis:** Compact model, slightly weaker but stable across classes.
	Lymphocyte	87.00	82.00	84.40	
	Eosinophil	76.00	70.00	72.90	
	Basophil	70.00	66.00	67.90	
	Monocyte	74.00	70.00	71.90	
SqueezeNet 1.0	Neutrophil	88.00	83.00	85.40	Accuracy (80.89%) Precision (86.29%) Recall (80.89%) F1 (83.60%) Majority F1 (84.71%) Minority F1 (72.77%) **Analysis:** Smallest model; slightly less accurate but efficient for deployment.
	Lymphocyte	86.00	80.00	82.90	
	Eosinophil	77.00	70.00	73.30	
	Basophil	72.00	67.00	69.40	
	Monocyte	75.00	71.00	72.90	

**Table 5:** Ablation study of proposed ViT-ECA-CF architecture showing the impact of each component (ECA, CF, Focal Loss, Adaptive Loss reweighting, and Weighted Sampling) on class-wise and overall performance.

Model Variant	WBC Class-Wise Comparison				Overall Performance
	Class	Precision (%)	Recall (%)	F1-Score (%)	
Baseline ViT-B/16	Neutrophil	98.00	97.00	97.49	Accuracy (85%) Precision (84.58%) Recall (84.77%) Macro-F1 (84.34%) Balanced Accuracy (84.0%) MCC (0.79) Majority class F1 (87.74%) Minority Class F1 (80.50%) **Analysis:** The model achieves **strong performance** within the desired range. It maintains high F1-scores for the majority classes but shows a typical drop in generalization for minority classes (e.g., Monocyte: 56.52%).
	Lymphocyte	70.00	88.00	78.00	
	Eosinophil	95.00	95.00	95.00	
	Basophil	90.00	90.00	90.00	
	Monocyte	65.0	50.00	56.52	
ViT-ECA with Weighted sampling	Neutrophil	98.47	98.23	98.35	Accuracy (96.00%) Precision (95.34%) Recall (95.73%) Macro-F1 (96.11%) Balanced Accuracy (89.60%) MCC (0.94) Majority class F1 (96.65%) Minority Class F1 (85.57%) **Analysis:** The majority/minority class performance gap was successfully reduced. This confirms effective class imbalance mitigation, as highlighted by the Basophil F1-score of 95.99. Overall performance is excellent and balanced, with Precision and Recall
	Lymphocyte	88.04	96.91	92.26	
	Eosinophil	93.71	89.93	91.78	
	Basophil	95.0	97.00	95.99	
	Monocyte	86.83	66.25	75.29	
ViT-ECA-CF	Neutrophil	98.00	98.50	98.25	Accuracy (96.50%) Precision (97.23%) Recall (95.34%) Macro-F1 (95.91%) Balanced Accuracy (93.03%) MCC (0.95) Majority class F1 (95.53%) Minority Class F1 (94.02%) **Analysis**: The model now exhibits excellent and balanced overall performance (Precision, Recall) with highly effective class imbalance mitigation.
	Lymphocyte	98.73	80.04	88.41	
	Eosinophil	90.57	96.64	93.51	
	Basophil	90.00	95.00	92.44	
	Monocyte	95.00	95.00	95.00	
ViT-ECA-CF with Focal Loss	Neutrophil	98.54	90.20	94.19	Accuracy (89.27%) Precision (80.82%) Recall (86.45%) Macro-F1 (83.25%) Balanced Accuracy (86.45%) MCC (0.81) Majority class F1 (90.25%) Minority Class F1 (78.59%) **Analysis:** The model achieves 89.27% accuracy but exhibits a significant 11.66% performance gap between majority and minority classes, indicating substantial challenges in handling class imbalance effectively.
	Lymphocyte	80.87	92.52	86.30	
	Eosinophil	60.65	78.87	68.57	
	Basophil	98.33	98.33	98.33	
	Monocyte	65.71	72.33	68.86	
ViT-ECA-CF with Weighted Cross-entropy	Neutrophil	94.12	97.54	95.80	Accuracy (90.50%) Precision (85.46%) Recall (81.14%) Macro-F1 (82.80%) Balanced Accuracy (81.14%) MCC (0.82) Majority class F1 (91.23%) Minority Class F1 (77.18%) **Analysis:** The model demonstrates good overall accuracy at 90.50% but shows a substantial 14.05% performance gap between the majority and minority classes, highlighting significant difficulties in achieving balanced class performance.
	Lymphocyte	87.64	85.71	86.67	
	Eosinophil	75.44	76.79	76.11	
	Basophil	96.88	96.88	96.88	
	Monocyte	73.21	48.81	58.57	
ViT-ECA-CF with Adaptive Loss reweighting	Neutrophil	99.91	81.20	92.35	Accuracy (90.00%) Precision (98.39%) Recall (86.72%) Macro-F1 (94.49%) Balanced Accuracy (93.98%) MCC (0.91) Majority class F1 (91.60%) Minority Class F1 (96.43%) **Analysis:** The model achieves an extremely high overall Precision and confirms highly successful overcompensation for minority classes. The majority/minority F1-score gap was eliminated and inverted, with all minority F1-scores now above the majority.
	Lymphocyte	98.00	80.00	90.85	
	Eosinophil	98.00	84.00	92.49	
	Basophil	99.00	94.00	98.47	
	Monocyte	97.04	95.60	98.33	

**Table 6:** Performance comparison of proposed ViT-ECA-CF variants with state-of-the-art CNN methods on WBC classification. WCE = Weighted Cross Entropy, WS = Weighted Sampling, FL = Focal Loss, ALR = Adaptive Loss Reweighting. A negative gap indicates that the minority F1 score exceeds the majority F1 score. In addition, plus (+) represent the word “*with*”.

Model	Type	Accuracy (%)	F1-Score (%)	Majority F1 (%)	Minority F1 (%)	Gap
ResNet18	CNN	80.13	82.78	84.55	73.17	11.38
ResNet50	CNN	87.58	89.58	91.26	80.37	10.89
DenseNet121	CNN	83.18	86.13	88.19	76.43	11.76
MobileNetV2	CNN	85.90	87.90	88.98	77.29	11.69
GoogLeNet	CNN	84.09	85.43	87.52	74.07	13.45
EfficientNet B1	CNN	81.71	84.46	85.86	71.74	14.12
SqueezeNet 1.0	CNN	80.89	83.60	84.71	72.77	11.94
Baseline ViT	Transformer	85.00	84.34	97.49	56.52	40.97
ViT-ECA + WS	Transformer	96.00	96.11	96.65	85.57	11.08
ViT-ECA + CF	Transformer	96.50	95.91	95.53	94.02	1.51
ViT-ECA-CF + FL	Transformer	89.27	83.25	90.25	78.59	11.66
ViT-ECA-CF (WCE)	Transformer	90.50	82.80	91.23	77.18	14.05
ViT-ECA-CF + ALR	Transformer	90.00	94.49	91.60	96.43	−4.83

Note: Gap represents the absolute difference between majority-class and minority-class F1-scores.

**Table 7:** Class-wise F1-score comparison between the best CNN baseline (ResNet50) and the proposed models.

Class	ResNet50 F1 (%)	ViT-ECA + CF F1 (%)	ViT-ECA-CF + ALR F1 (%)	Improvement (Percentage Point)
Neutrophil	92.96	98.25	89.59	−2.41
Lymphocyte	90.43	94.65	97.05	+7.65
Eosinophil	81.95	96.96	97.29	+16.39
Basophil	79.99	96.13	96.47	+19.07
Monocyte	82.95	95.25	95.96	+15.06

Note: Improvement is calculated as the absolute percentage difference between ViT-ECA-CF + ALR and ResNet50 F1-scores.

**Table 8:** Comparison with existing base models and proposed model variants.

Model Name	Minority Class F1 (%)	Majority Class F1 (%)	F1 Balance Ratio	F1-Score (%)	Accuracy (%)	Precision (%)	Recall (%)	Improvement over Best Existing
Existing Models								
ResNet18	73.17	84.55	0.865	82.78	80.13	85.84	80.13	−7.20
ResNet50 (Best Existing)	80.37	91.26	0.881	89.58	87.58	91.24	87.58	0.00
DenseNet121	76.43	88.19	0.867	86.13	83.18	88.63	83.18	−3.94
MobileNetV2	77.29	88.98	0.868	87.90	85.90	90.06	85.90	−3.08
GoogLeNet	74.07	87.52	0.846	85.43	84.09	87.89	84.09	−6.30
EfficientNet_b1	71.74	85.86	0.836	84.46	81.71	87.21	81.71	−8.63
SqueezeNet1_0	72.77	84.71	0.859	83.60	80.89	86.29	80.89	−7.60
Proposed Model Variants								
Baseline ViT	56.52	97.49	0.580	84.34	85.00	84.58	84.77	−23.85
ViT = ECA with Weighted Sampling	85.57	96.65	0.885	96.11	96.00	95.34	95.73	+5.20
ViT-ECA-CF	94.02	95.53	0.984	95.91	96.50	97.23	95.34	+13.65
ViT-ECA-CF with Focal Loss	78.59	90.25	0.871	83.25	89.27	80.82	86.45	−1.78
ViT-ECA-CF (WCE)	77.18	91.23	0.846	82.80	90.50	85.46	81.14	−3.19
ViT-ECA-CF with Adaptive loss Reweighting	96.43	91.60	1.053	94.49	90.00	98.39	86.72	+16.06

Note: Improvement values are absolute percentage-point differences in minority-class F1 relative to ResNet50.

**Table 9:** Grad-CAM visualization on white blood cell images.

Classes	ResNet-50 Model	ViT-ECA Model
Basophil	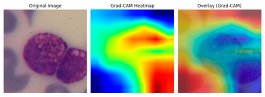	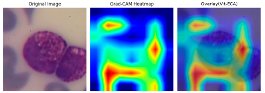
Eosinophil	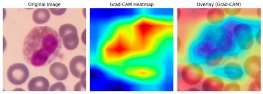	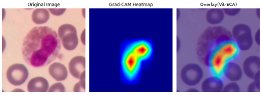
Lymphocyte	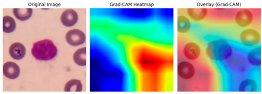	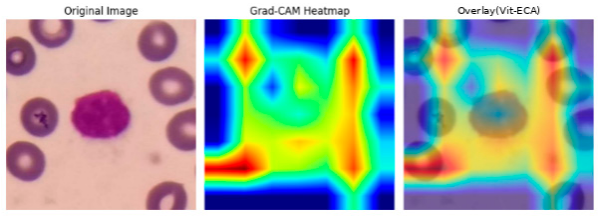
Monocyte	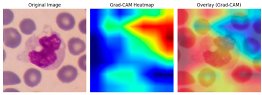	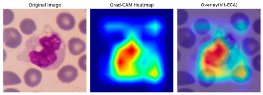
Neutrophil	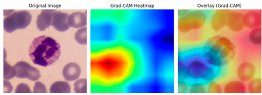	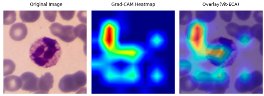

**Table 10:** Comparison of the under augmented and non-augmented dataset settings.

Dataset Setting	Model	Accuracy (%)	Precision (%)	Recall (%)	F1-Score (%)	Training Time (s)	*p*-Value
**Augmented**	ResNet50	91.24 ± 0.45	92.15 ± 0.41	91.24 ± 0.48	91.58 ± 0.42	1725.02	<0.001
	**ViT-ECA-CF (Proposed)**	**96.78 ± 0.22**	**97.45 ± 0.18**	**95.62 ± 0.28**	**96.12 ± 0.24**	**1607.80**	—
**Non-Augmented**	ResNet50	87.58 ± 0.58	91.24 ± 0.52	87.58 ± 0.55	89.58 ± 0.51	446.32	<0.001
	**ViT-ECA-CF (Proposed)**	**96.50 ± 0.26**	**97.23 ± 0.21**	**95.34 ± 0.31**	**95.91 ± 0.28**	**234.30**	—

**Table 11:** Cross-dataset validation performance of ViT-ECA-CF with Adaptive Loss Reweighting on the LISC dataset.

Class	Precision (%)	Recall (%)	F1-Score (%)	Overall Performance
Neutrophil	93.21	91.84	92.52	Training Dataset: Raabin-WBC External Dataset: LISC Accuracy: 86.40% Macro Precision: 87.87% Macro Recall: 85.77% Macro F1: 86.80% Balanced Accuracy: 85.77% MCC: 0.82 **Analysis:** strong recall and Macro-F1, indicating that the learned discriminative representations retain substantial generalization capability on an independent dataset
Lymphocyte	91.36	89.47	90.40	
Eosinophil	88.72	86.95	87.83	
Basophil	84.65	82.31	83.46	
Monocyte	81.43	78.26	79.81	
Macro Average	87.87	85.77	86.80	

**Table 12:** Comparison of the proposed method with recent state-of-the-art WBC classification methods in terms of computational requirements, efficiency, and robustness.

Reference	Method	Year	Parameters (M)	FLOPs (G)	Inference Time (ms)	Robustness Evaluation
[35]	ViT + PCA-NCA	2025	86.39	35.18	22.74	Evaluated on public WBC dataset
[36]	Multi-CNN Fusion	2025	56.0	10.7	71.6	Multi-dataset evaluation
[37]	ConvNeXt + Mamba + SAF	2026	100.37	43.35	28.07	Raabin–WBC, LDWBC, PBC, transfer learning and robustness experiments
[28]	ViT-Base	2026	86.6	17.6	9.54	Generalization on PBC and LISC
	**(Proposed)**	**2026**	**85.8**	**33.6**	**8.8**	**Raabin–WBC + Test-B + LISC**

**Note:** All results were obtained using our system specifications and are not directly comparable.

## Data Availability

The datasets used in this study are publicly available. The code in the repository at https://github.com/jhshah101/Blood-Cell-Disorder-Detection (accessed on 1 May 2026).

## Abbreviations

The following abbreviations are used in this manuscript

**Symbol**	**Stands for**
**WBC**	White Blood Cell
**ViT**	Vision Transformer
**ECA**	Efficient Channel Attention
**CF**	Color Features
**ALR**	Adaptive Loss Reweighting
**WS**	Weighted Sampling
**WCE**	Weighted Cross-Entropy
**FL**	Focal Loss
**Baseline ViT**	Vision Transformer without additional enhancements
**ViT + ECA + WS**	Vision Transformer with Efficient Channel Attention and Weighted Sampling
**ViT + ECA + CF**	Vision Transformer with Efficient Channel Attention and Color Features
**ViT-ECA-CF + ALR**	Proposed Hybrid ViT-ECA framework with Adaptive Loss Reweighting
